# Natural polymer derivative-based pH-responsive nanoformulations with entrapped diketo-tautomers of 5-fluorouracil for enhanced cancer therapy

**DOI:** 10.5599/admet.2554

**Published:** 2025-01-03

**Authors:** Anbazhagan Thirumalai, Koyeli Girigoswami, Karthick Harini, Venkatakrishnan Kiran, Pazhani Durgadevi, Agnishwar Girigoswami

**Affiliations:** Medical Bionanotechnology, Faculty of Allied Health Sciences, Chettinad Hospital & Research Institute (CHRI), Chettinad Academy of Research and Education (CARE), Kelambakkam, Chennai, TN-603103, India

**Keywords:** Oxidized sodium alginate, polymeric nanoparticles, anticancer activity, pH-responsive drug delivery

## Abstract

**Background and purpose:**

Despite significant advancements in cancer therapies, chemotherapeutics continue to be the mainstay for treating cancer patients, with 5-fluorouracil (5-FU) being commonly used for various cancers. However, its limited ability to penetrate cell membranes and its short half-life, caused by rapid metabolism, necessitate frequent administration of high doses to maintain effective therapeutic levels. This study aimed to synthesize oxidized sodium alginate (OSA) derivatives to create OSA nanoparticles loaded with 5-FU (OSANP@ 5-FU), promoting diketo tautomers, and evaluate their photophysical properties, release profile, and anticancer activity with minimal toxicity.

**Experimental approach:**

The investigation encompassed physicochemical characterization, encapsulation efficiency, 5-FU release kinetics at pH 2.2 and 7.4, cell viability assessment via MTT assay in V79 cells, and in vitro anticancer efficacy in the A375 cell line.

**Key results:**

Steady-state absorption and emission confirmed the presence of advantageous diketone tautomers of 5-FU, indicating radiative transitions from the second singlet excited state to the ground state (S2→S0) and the drug's encapsulation within the polymeric nanostructure. Dynamic light scattering revealed that OSA nanoparticles, initially 177.8 nm, grew to 226.6 nm after encapsulating 5-FU, retaining high zeta potential for stability. With a 68% encapsulation efficiency, in vitro studies showed 46 to 54 % of 5-FU released across different pH levels within 510 minutes.

**Conclusion:**

In acidic conditions, there is a greater release of 5-FU than neutral pH levels, indicating a pH-responsive release profile beneficial for cancer treatment, with the release mechanism of OSANPs following Fickian diffusion as identified by a Korsmeyer-Peppas mathematical model and the formulation showing improved therapeutic efficacy.

## Introduction

Despite the significant advances in medicine and technology, cancer continues to be the second leading origin of death internationally, with low-income as well as middle-income countries bearing the brunt of this disease, accounting for approximately 70 % of all cancer-connected deaths all-inclusive [1,2]. In India, although the number of reported new cancer cases in 2020 was lower at 1.32 million compared to 2.28 million in the USA, the death toll was significantly higher, with approximately 850,000 deaths in India versus 610,000 in the USA, as per the World Health Organization report [3,4]. This progress in cancer biology and genetics is evident through the discovery of over 1,000 genes that undergo alterations in tumors due to genetic mutations or epigenetic modifications [5]. Such advancements enable detailed mapping of molecular pathways, cellular structures, and the behavior of mutated cancer cells, providing insights into tumor development and spread [6]. However, while therapeutic approaches based on these findings offer benefits for certain cancer types, they often encounter challenges such as tumor resistance, which restricts their effectiveness in improving overall survival rates. Cancer treatments, including surgery, chemotherapy, immunotherapy, and radiotherapy, can be employed individually or in combination, contingent upon the type and stage of cancer, each carrying its benefits and drawbacks [7-9]. Despite advancements in developing new strategies for cancer treatment, chemotherapy remains a primary approach to managing the disease of cells. Because of their lack of precision and increased toxicity, the clinical use of chemotherapeutic drugs has been constrained, with intravenous administration often utilized to maximize drug absorption and distribution throughout the body [10]. However, the effectiveness of many chemotherapeutic agents is still hindered by factors such as shorter circulation half-life, insufficient penetration into tumor tissues, and the diverse characteristics of tumors [11]. The primary challenge in treatment failure lies in the drug's inability to reach the target site, compounded by the lack of specificity, causing side effects on both healthy and tumor cells. Additional drawbacks of anticancer drugs include hydrophobicity, susceptibility to resistance development, and inadequate biodistribution. Increasing the dosage or frequency of administration to achieve therapeutic levels often results in heightened toxicity [12,13].

5-fluorouracil (5-FU) is a derivative of uracil, where a fluorine atom replaces a hydrogen atom at the C-5 position [14]. It enters cells quickly through the same enabled transport mechanism as uracil [15]. As an antimetabolite drug, 5-FU is known to disrupt the integrity of macromolecules such as DNA and RNA by inhibiting crucial biosynthetic processes or being incorporated into them to disrupt their normal function [16-18]. 5-FU has broad-spectrum anticancer activity and can be used independently or as part of combined chemotherapeutic treatments [19]. 5-FU prompts cytotoxicity by hindering thymidylate synthase-driven biosynthetic processes and integrating its metabolites into RNA and DNA, leading to transcription and replication errors, activation of DNA damage response pathways, and apoptosis. The limited therapeutic effectiveness due to the short half-life and reduced affinity for tumor cells necessitates higher drug dosages to achieve desired results, albeit at the cost of increased toxicity [20]. Researchers have used an alternative strategy to enhance the bioavailability and affinity of drugs toward tumors by using nanocarriers for the targeted delivery of tumors. In recent times, nanocarrier-mediated drug delivery systems have been widely employed to improve the efficacy of anticancer drugs by addressing issues such as aqueous insolubility, low selectivity, poor stability, high toxicity, and side effects [21]. Many studies show that anticancer drugs loaded into the nano-vesicles significantly enhance treatment efficiency and reduce drug side effects compared with free drugs [22]. Anjum *et al.* [23] developed nanoparticles using thiolated chitosan and coated them with hyaluronic acid to specifically deliver 5-FU, aiming to enhance its effectiveness. Khan *et al.* [24] created hybrid lipid-PLGA nanoparticles containing 5-FU through a three-factor and three-level Box-Behnken design, focusing on achieving sustained release and improved anticancer efficacy in vitro. Li *et al.* [25] described a well-planned and eco-friendly synthesis of lysozyme-hyaluronan composite colloidal nanoparticles, which are functionalized and co-loaded with 5-FU and curcumin. These nanoparticles aim to provide more effective targeted therapy for colorectal cancer while reducing side effects [26,27]. Manivannan et al. encapsulated 5-FU and docetaxel in chitosan nanoparticles and showed that the combined effect of these drugs within the nanoparticles was significantly greater in cancer cells compared to the effect of each drug alone [28]. Nanostructures are highly favored as chemotherapy drug delivery systems due to their affordability, compatibility to human body, and non-toxic nature, particularly excelling as colloidal carriers [29-31].

Various nanocarriers, including liposomes, dendrimers, polymer nanoparticles, micelles, carbon nanotubes, and inorganic nanoparticles, hold promise for drug delivery [32-36]. Polymeric nanoparticles, in particular, have emerged as a focus of extensive research due to their capacity to transport diverse drugs effectively and provide controlled release mechanisms [37-39]. The chemical composition of polymeric nanoparticles can be customized to match the chemical structure of drugs, aiming to optimize compatibility between the carrier and the drug [40-42]. Numerous nanosystems are employed in drug delivery systems, with polysaccharide-based nanocarriers, such as alginate, playing a crucial role in delivering multiple anticancer drugs due to their beneficial properties and successful outcomes [43,44]. Derived from brown seaweeds, alginate comprises α-L-guluronate and β-D-mannuronate monomers linked by 1,4-glycosidic bonds, with variations in monomer ratios dependent on the seaweed species, offering high biocompatibility, biodegradability, and non-toxicity [44]. Alginate's ability to dissolve under neutral and alkaline pH conditions, influenced by its carboxyl groups becoming charged around pH 4, supports its extensive use. It is a favored polymer for pharmaceutical applications where drugs need shielding or specific absorption in the intestines and sustained or controlled drug release. Sodium alginate, widely employed in pharmaceuticals, extends drug release by altering its chemical properties and viscosity levels. In acidic environments, protonation of alginate's carboxyl groups limits drug release [45]. According to Bouhadir et al., alginate can be oxidized with periodate to produce oxidized sodium alginate (OSA), a compound with multiple functional aldehyde groups [46]. OSA can be further modified chemically or physically to fine-tune its properties for specific drug delivery applications.

**Scheme 1.**
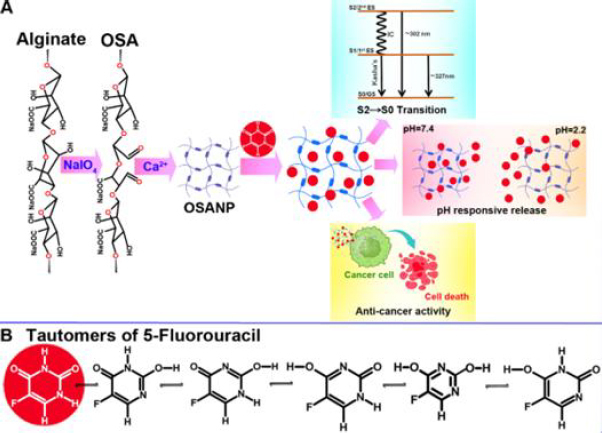
**(A)** Pictorial depiction of the strategy and the output of the study. **(B)** possible tautomeric forms of 5-FU

Polymer-based nanotechnology for drug delivery offers significant promise in transforming how drugs are delivered, ensuring precise and effective therapeutics targeting infection sites while minimizing impacts on healthy tissues [47,48]. Our current research is centered on developing biodegradable OSA nanocarriers designed to improve the pH-dependent effectiveness of therapeutics (Scheme 1). OSA has been synthesized and modified to encapsulate the anticancer drug 5-FU (Scheme 1A). This was followed by comprehensive physicochemical characterization and evaluation of anticancer activity using the A375 cell line to establish efficacy and pH-responsive release profiles with reduced toxicity. Mathematic modeling was used to establish 5-FU release kinetics.

## Experimental

### Materials

Sodium alginate (Alg), calcium chloride, sodium periodate (NaIO_4_), sodium chloride, thiazolyl blue tetrazolium bromide (MTT), and ethylene glycol were acquired from Himedia, India. Acetone and ethanol were obtained from Sigma, India. Fetal bovine serum (FBS), trypsin-EDTA, and antibiotic solutions were obtained from Gibco, USA. All the chemicals utilized in this experiment were used as procured, without additional purification, and double distilled sterile water (d/w) was employed throughout the experiment. A375 and V79 cells were acquired from the National Centre for Cell Science (NCCS), India.

### Characterization

The functional groups of the synthesized nanoparticles were investigated using a Bruker-Alpha FTIR/ATR spectrometer. A Shimadzu UV-1800 spectrometer was used to measure the absorption spectra. Jasco FP-3800 spectrofluorometer was used to measure excitation and emission spectrum. Malvern Nano-ZS-90 nanoparticle size analyzer was used to investigate the hydrodynamic diameter (dH) and zeta potential using the dynamic light scattering principle. The surface etiology of the synthesized nanoparticles was analyzed by FEI Quanta 200FEG scanning electron microscope (SEM). Fluorescent cellular images were captured using the Olympus BX-51 fluorescence microscope. Olympus CKX41 inverted microscope used to take live cellular imaging.

### Preparation of oxidized sodium alginate

To prepare sodium alginate (OSA), an equal ratio of Alg and NaIO_4_ was liquefied in 200 ml of d/w and kept stirring for 20 hours in a dark condition. Ethylene glycol (0.7 ml) was supplemented with the above mixture and stirred for 30 min. Then, 0.6 g of sodium chloride was dissolved in the mixture, followed by the addition of 200 ml of ethanol to obtain OSA precipitate, and the mixture was separated by centrifugation at 4500 rpm for 10 min. To procure pure OSA, the obtained precipitate was redispersed in 100 ml d/w, mixed 0.1 g of sodium chloride with 200 ml acetone, and kept undisturbed. After 40 minutes, the precipitant was separated by removing the top layer, and the corresponding step was repeated thrice. Then air dried the precipitant at room temperature (25 ± 3°C) to obtain a white-colored pure OSA powder [49,50].

### Synthesis of 5-FU encapsulated OSA nanoparticles

The ionic gelation method was used to develop oxidized sodium alginate nanoparticles (OSANPs). 0.075 g of OSA were dissolved in 25 ml d/w; 5 ml of 30 mM calcium chloride was added dropwise in a stirring condition at 500 rpm and continued stirring for 30 mins. The transition to opalescent colloidal mixture indicates the formation of OSA nanoparticles. To generate a uniform size distribution, the suspension was undisturbed overnight and confirmed by measuring the hydrodynamic diameter. The synthesized nanoparticles were centrifuged for 20 min at 3500 rpm, and the pellet was redispersed in d/w to remove the unreacted ingredients; the procedure was reiterated, and finally, the pellet was dispersed in 25 ml PBS (pH 7.4). The 5-FU was loaded into OSA nanoparticles by sonicating 5-FU and nanoparticles together in a water bath sonicator for 5 mins.

### Encapsulation efficiency and release kinetics

The encapsulation efficiency (EE) of 5-FU entrapped in OSANPs was calculated spectrophotometrically by using Equation (1) below. The outer diameter (OD) of the 5-FU-loaded OSANPs (*A*) was taken at 265 nm. The 5-FU-loaded OSANPs were centrifuged at 15000 rpm for 15 min, and the supernatant was collected to analyze unencapsulated or free 5-FU (*B*).



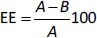

(1)


The release kinetics of 5-FU from OSA nanoparticles were analyzed in vitro using the dialysis bag diffusion method, with studies conducted in both an HCl/KCl buffer (acidic medium at pH 2.2) and a PBS buffer (neutral medium at pH 7.4). 2 ml of 5-FU-encapsulated OSA nanoparticles were enclosed in a dialysis membrane and placed in 50 milliliters of buffer solution. The release of 5-FU was monitored over 510 minutes at regular intervals using spectrophotometric analysis, with measurements taken at 265 nm.

The Korsmeyer-Peppas model was used to determine the drug transport mechanism for OSANPs. This, also known as the "Power law" model, is used to explain how drugs are released from a polymeric system in situations where the exact release mechanism is unclear or when multiple release phenomena are at play. This model accounts for several simultaneous release mechanisms, including water diffusion into the matrix, dissolution, and swelling of the matrix [51]. The 5-FU release from the OSA nanoparticles over time was also graphed using the Korsmeyer-Peppas model, described by a specific Equation (2).



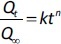

(2)


Here, *Q*_t_ represents the amount of drug released at time (*t*), and *Q_>_* is the total amount released at infinite time (). The constant *k*, which includes the structural and geometric characteristics of the drug delivery system, and *n*, the release exponent, indicate the drug release mechanism from the polymer nanoparticle.

### In vitro anticancer assessment

The anticancer activity of the produced OSANP@ 5-FU was determined in assessment with the same concentration of free 5-FU and OSANPs encapsulated 5-FU by treating with A375 cells. A375 cells were seeded by a density of 2×10^5^ cells each well in 24-well culture plates containing DMEM complete medium, 10 % FBS, and antibiotic solutions. The cells were nurtured in a moisturized environment with 5 % CO_2_ at 37 °C for 24 hours. Then, these cells were treated with increasing concentrations (2-20 μg/ml) of 5-FU and OSANP@ 5-FU and further gestated for 24 h. Thereafter, 100 μl of MTT salt solution (5 mg/ml concentration) was introduced under dark conditions and continued incubation for 4 h. DMSO was added to liquefy the formazan crystals formed by live cells, and the OD was recorded at 570 nm.

### Live dead assay

A double staining method was used to evaluate the cells' viability, and fluorescent images of live and dead cells were captured. In a coverslip, A375 cells (1.5×10^5^) were cultivated in DMEM complete medium by 24 h incubation at 37 °C at humidified conditions. Subsequently, the cells were treated with 20 μg/ml of 5-FU and OSA NPs- 5-FU and further incubated for 24 h. The coverslip was then sited on a grease-free and germ-free glass slide, and a mixture of acridine orange and ethidium bromide (100 μg/ml) dyes was added and further incubated at 37 °C for 3 min. The stained cells were visualized and pictured under a fluorescent microscope using an appropriate filter. The rate of deceased cells, %, was calculated using a specific Equation (3)



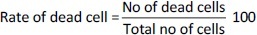

(3)


### Scratch assay

The A375 cells were maintained in a DMEM complete medium, and cell seeding was done on 35 mm petri plates and incubated at 37 °C, 5 % CO_2_ in a humidified environment allowed to reach 75 to 90 % confluence. A consistent scratch was created in each plate using a sterile 10 μl pipette tip. After removing the debris by washing the cells with sterile PBS, they were exposed to 20 μg/ml of 5-FU and OSANP@ 5-FU for 24 and 48 h, following which cell images were captured using an inverted microscope.

### Toxicity assessment

The cytotoxicity of OSA nanoparticles was estimated by performing an MTT assay, following the method of Harini *et al*. [34] with minor adjustments. In brief, V79 cells were cultivated in DMEM complemented with 10 % FBS and 1 % antibiotics for 24 h at 37 °C in a moistened environment with 5 % CO_2_. Approximately 1.5×10^4^ V79 cells were plated in each well of 24 well plates and were kept for additional incubation for 24 h. Cell treatment was done with 5, 10, 20, 30 and 40 μg/ml of OSANPs and incubated under similar growth conditions for 24 h. Subsequently, 100 μl of 5 mg/ml MTT solution was added to every well in the dark, and the plates were kept in the incubator for 4 h with the same conditions. DMSO was added to dissolve the formazan crystals formed by live cells, and the OD was recorded at 570 nm. Cell feasibility or viability, %, was determined using Equation (4):



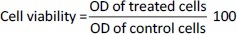

(4)


## Results and discussion

### Physicochemical characterization

**Figure 1.**
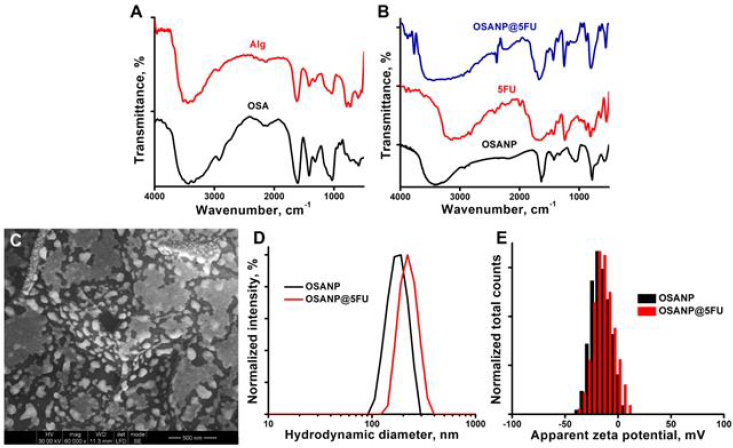
**A** - FTIR spectra of alginate and OSA; **B** - FTIR spectra of OSANPs, 5-FU, and OSANP@ 5-FU; **C** - SEM image of OSA NPs; **D** - colloidal particle size distribution of OSANPs and OSANP@ 5-FU; **E** - Zeta potentials of OSANPs and OSANP@ 5-FU.

The alginate chain features adjoining hydroxyl (-OH) groups at the second (C-2) and third carbon (C-3) positions, which can be selectively oxidized by periodate to form aldehyde groups. Sodium periodate is the most commonly used oxidizing agent for introducing active functional moiety and enabling easy transformation of sodium alginate in controlled drug delivery systems. When treated with sodium periodate, the C2–C3 bond is cleaved, converting uronic acid into a nearly linear or open-chain structure with aldehyde (-CHO) groups. These aldehyde groups can interact with adjacent hydroxyl groups (-OH) on the alginate chain to create cyclic hemiacetals. This oxidation process greatly reduces the molecular weight of sodium alginate, leading to the formation of highly reactive aldehyde groups. The oxidized sodium alginate maintains improved water solubility, lower toxicity, and higher alginate biocompatibility while offering better biodegradability and increased molecular flexibility. Understanding the correlation between the oxidation degree of OSA and its biodegradability and gelation properties is essential for exploring the potential of OSA-based hydrogels in carrying 5-FU, an anticancer agent. Figure 1A displays the FTIR spectra of Alg (sodium alginate) and OSA. For alginate, the general characteristic band at 3445 cm^-1^ corresponded with the stretching of –OH groups, and a minor intensity band at 2141 cm^-1^ was linked to –CH_2_ groups. The asymmetric and symmetric stretch at 1615 and 1414 cm^-1^ are the characteristic carboxylate (_-_COO^-^) vibrational modes. The vibrational range between 1038 and 790 cm^-1^ is assigned to the C-O-C symmetrical stretching of the acetyl group in alginate. Furthermore, a new weak band at 1726 cm^-1^ corresponded to the C=O stretching of aldehyde groups. The specific bands at 948 cm^-1^ and 726 cm^-1^ indicate the –CH out of the plane bending. Observation of these representative bands suggests that OSA was successfully synthesized.

Figure 1B demonstrates FTIR spectra of OSANPs and 5-FU encapsulated OSANP or OSANP@5-FU. The band at 3423 cm^-1^ corresponded to –OH stretching vibration groups, polymeric association, and intermolecular hydrogen bond. The bands at 1634 and 1425 cm^-1^ demonstrated the asymmetrical and symmetrical stretching of –COO groups. A weak band at 1745 cm^-1^ demonstrated the C=O stretching of the aldehyde group. The band at 1074 cm^-1^ is correlated with C-O stretching vibration, while the band at 576 cm^-1^ is due to C-H out-of-plane bending. The narrower -O=H stretching vibration band in calcium alginate arises from the chelating structure formed by calcium ions binding to hydroxyl groups. Conversely, the broader peak in oxidized sodium alginate results from the presence of carbonyl groups. After encapsulation, the new band at 796 and 1364 cm^-1^ is because of the FC=CH groups and aromatic vibration. The broad band at 3547 to 2835 cm^-1^ is due to the presence of more -C-F groups (unbound) from the 5-FU greatly shows the successful encapsulation of 5-FU into OSA nanoparticles.

The surface morphology of the OSANPs was characterized using SEM analysis. Figure 1C shows the successful formation of OSANPs, and the particles are slightly elongated-spherical in shape and regularly distributed. The mean diameter of the OSANPs was calculated as 89.22±12.2 nm. A particle size analyzer was used to examine the colloidal properties in terms of the hydrodynamic nature of synthesized OSANPs and 5-FU encapsulated OSANPs (OSANP@ 5-FU) in a Malvern Nano-ZS-90 nanoparticle size analyzer. The dH of the OSANPs was measured as 177.8 nm, while the OSANP@ 5-FU exhibited a dH of 226.6 nm, as depicted in Figure 1D. The polydispersity index (PDI), which indicates the distribution of particle sizes, was 0.478 for the OSANPs and 0.363 for the OSANP@5-FU, respectively. The PDI value indicates the synthesized OSANPs and 5-FU encapsulated OSANPs are monodispersed in nature [52]. The zeta potential for the OSANPs was recorded as -15.8 mV, indicating a stable colloidal system. In comparison, the OSANP@5-FU showed a slightly lower –ve value of zeta potential of -14.2 mV, as shown in Figure 1E. The measurement of zeta potential is crucial as it reflects the stability of nanoparticles, with the surface charge affecting their behavior in suspension. The changes in hydrodynamic diameter and zeta potential values of the OSANPs and OSANP@ 5-FU are due to the changes in their microenvironment, which confirms the encapsulation of 5-FU into OSANPs.

### Optical spectroscopic observations

**Figure 2.**
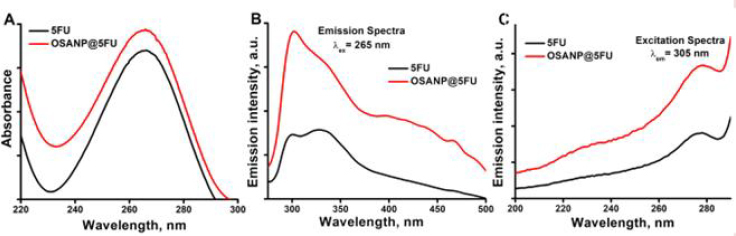
**A** - Absorption spectra of 5-FU and OSANP@ 5-FU; **B** - Emission spectra of 5-FU and OSANP@ 5-FU at λ_ex_ = 265 nm; **C** - Excitation spectra of 5-FU and OSANP@ 5-FU at λ_em_ = 305 nm.

The absorption spectra of 5-FU and OSANP@ 5-FU were measured with the UV-visible spectrophotometer to ensure successful encapsulation of 5-FU into the OSANPs. The maximum absorption band (λ_max_) of 5-FU was observed at 265 nm, as shown in Figure 2A. The absorption spectra of OSANP@ 5-FU showed a greater absorption band or optical density (OD) at 265 nm for the same concentration of 5-FU. The OSA matrix provides a protective environment that can enhance the stability of 5-FU and protect it from degradation, leading to increased absorption. Our hypothesis suggests that changes in the absorption band are influenced by the molar extinction coefficient and the microenvironment created by drug encapsulation. Steady-state fluorescence measurements offer a greater sensitivity compared to spectrophotometric measurements. The steady-state fluorescence is employed to confirm drug encapsulation, as the fluorescent properties of the molecule may alter after encapsulation (Figure 2B, C). 5-FU showed an unstructured emission peak upon exciting at the λ_ex_ = 265 nm with an emission range of 275 to 500 nm. 5-FU predominantly exists in four tautomeric forms, namely diketo, keto-enol, and dienol, with diketo being the most stable, followed by keto-enol and dienol in descending order of stability [53]. Therefore, in aqueous solutions, 5-FU primarily exists in the 2,4-dioxo form [54]. An aqueous solution of 5-FU exhibited a broad emission with two wave-like peaks at 300 and 327 nm (Figure 2B). The peak at 300 nm is likely due to the radiative transition of the diketone tautomer of 5-FU (Scheme 1B) from the second singlet excited state to the ground state (S2→S0), while the peak at 327 nm can be attributed to the S1 to ground state transition (S1SS0) [55]. Additional humps at higher wavelengths in the spectrum are caused by the emission of minor keto-enol tautomers of 5-FU (Scheme 1B). Interestingly, the encapsulated 5-FU in OSA hydrogels displayed a distinct emission peak at ~302 nm with a small shoulder, likely due to the potent S2→S0 transition, indicating the predominant encapsulation or binding of 5-FU to the OSA hydrogels. The detection of radiative emission from the S2 state to the S0 state clearly breaches Kasha’s rule, which posits that the emission takes place from the lowest electronic excited state (S1) of the given multiplicity, irrespective of the excitation wavelength. The emission intensity also became 2.5-fold higher due to this entrapment, which can be useful in theranostic applications. The excitation spectra (Figure 2C) revealed a distinct peak at 278 nm, which differs from the absorption maximum at 265 nm, indicating a transition from the second electronic excited state (S2) to the ground state (S0), while the S1→S0 transition remained unidentified. The increased intensity of 5-FU in OSA nanostructures confirms successful encapsulation and improved photophysicochemical properties.

### Stability studies

**Figure 3.**
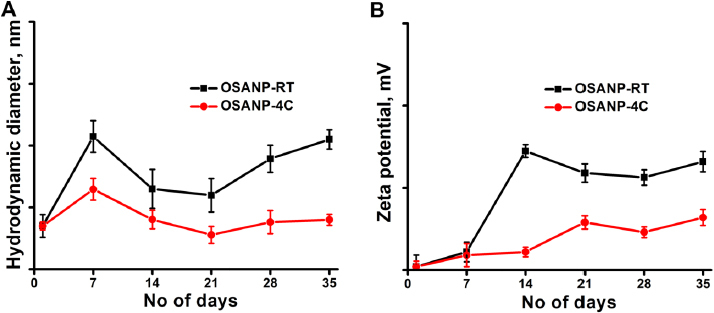
The stability of the OSA NPs was investigated by the hydrodynamic size (**A**) and zeta potential (**B**) for 5 weeks at 4 °C and room temperature (30±3 °C).

The stability of the synthesized OSA nanoparticles was assessed based on the impact of dH and surface charge. The nanoparticles were subjected to incubation at room temperature (RT= 30 °C) and 4 °C for a duration of 5 weeks, with regular monitoring for any observed changes. Figures 3 illustrate the dH and surface charge changes. In contrast, storing OSANPs at 4 °C showed no notable changes compared to room temperature, highlighting their high stability at lower temperatures. These findings affirm that the synthesized OSA nanoparticles maintain their structural integrity and properties exceptionally well when stored at 4 °C over an extended period, underscoring their suitability for biomedical applications.

### Drug encapsulation, release kinetics and cell viability assay

**Figure 4.**
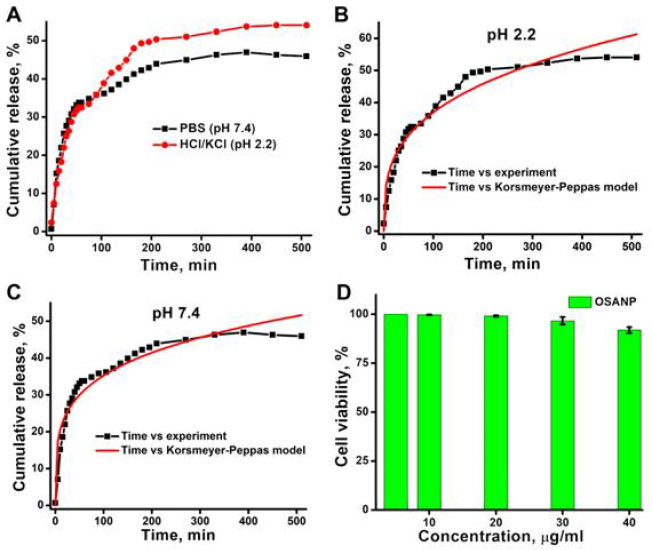
**(A)** Cumulative 5-Fluorouracil release profile from the OSANPs in two different pH. **(B)** Korsmeyer-Peppas is exemplary of the release profile of 5-FU from OSANPs in pH 2.2. **(C)** Korsmeyer-Peppas model to the release profile of 5-FU from OSANPs in pH 7.4. **(D)** Cell viability study using MTT assay on V79 cell line.

The encapsulation efficiency (EE) of OSANP@ 5-FU was calculated spectrophotometrically by recording the OD of the drug 5-FU at 265 nm and was found to be 68 %. Figure 4A shows the cumulative release of 5-FU from OSANPs in different release mediums (pH 2.2 and 7.4) for 510 minutes. It was observed that the release of 5-FU was 54 % in acidic pH and 46.8 % in neutral pH from OSANPs, indicating better release in acidic conditions. Initially, a rapid release was noted, likely due to loosely bound drug molecules on the surface of the nanoformulations. This was followed by sustained discharge of 5-FU over time. The increased release of 5-FU in an acidic environment makes it particularly effective for targeting cancer cells, which are often more acidic than normal cells. This pH-receptive drug transport system is promising for enhancing cancer treatment by improving the meticulousness of drug delivery, thereby increasing its effectiveness while simultaneously reducing potential side effects. Such a system ensures that the drug is released primarily in the vicinity of cancer cells, sparing healthy tissues and minimizing harmful consequences.

The release kinetics data were examined by applying the Korsmeyer-Peppas model to provide a detailed understanding of the drug release mechanism. This model helps to identify the nature of the release process, whether it is controlled by diffusion, erosion, or a combination of both, offering valuable insights into the behavior of the drug delivery system. The flowing plots were made, which are presented in Figures 4B and 4C are *Q_t_*/*Q_∞_ vs. t*. The *r*, *k*, and *n* values found for the Korsmeyer-Peppas model are shown in Table 1 for the two release mediums (pH 2.2 and 7.4) of 5-FU in OSANPs. When the value of *n* is lesser than or similar (≤) to 0.34, it suggests that the release mechanism follows Fickian diffusion, also known as Case I transport. Conversely, when the value of *n* is 0.85 or higher, the release mechanism shadows the case II transport. This mechanism involves the puffiness and relaxation of the polymeric matrix, which is a non-Fickian diffusion process. An intermediate value of n where (0.43 < *n* < 0.85) specifies an irregular (non-Fickian) transport mechanism. This proposes that the drug discharge is controlled by more than one process: the Fickian diffusion of the drug molecules and the erosion or relaxation of the polymer matrix. The *n* value for the release of 5-FU from OSA nanoparticles was 0.31 at pH 2.2 and 0.23 at pH 7.4, indicating that the Fickian diffusion mechanisms are at play when n is less than or equal to 0.34. This indicates that the drug release is primarily governed by the diffusion process, which is directly related to the concentration gradient of the drug within the polymer matrix. Fickian diffusion implies that the rate of drug release is much slower than the degree of polymer easing, and the drug molecules migrate through the matrix at a rate determined by Fick's laws of diffusion.

**Table 1:** The correlation coefficient (*r*), release kinetic constant (*k*), and release exponent (*n*) were calculated for the Korsmeyer-Peppas model to describe the release of 5-FU from OSA nanoparticles across different release media.

Release medium	*r*	*k*	*n*
pH 2.2	0.972	8.83	0.31
pH 7.4	0.964	11.98	0.23

To determine the suitability of OSANPs as a drug delivery system, the cytotoxicity of the nanoparticles was evaluated using MTT assay on V79 (fibroblasts) cell lines (Figure 4D). The OSANPs showed negligible toxicity (more than 90 % of cells are viable) after treatment with varying concentrations (5 to 40 μg/ml). The results suggest that the OSANPs can be used as a carrier to deliver drugs.

### Examination of the anticancer activity

**Figure 5.**
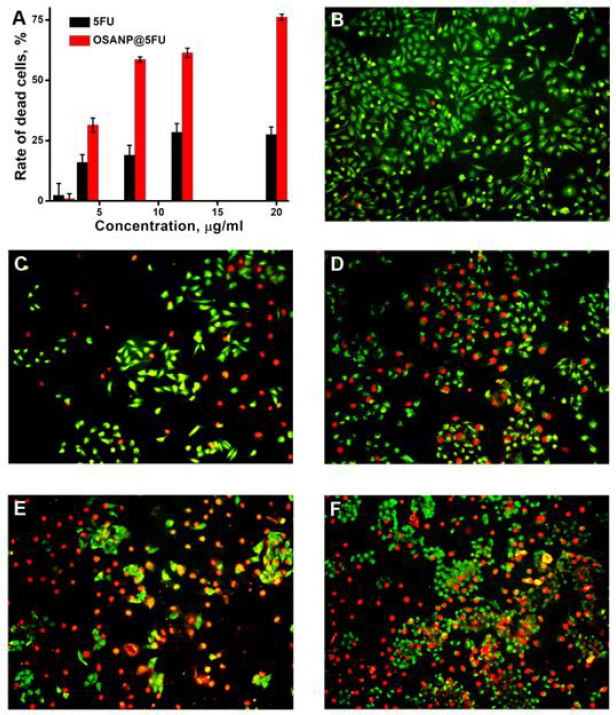
**A** - Rate of dead cells after treatment with 2, 4, 8, 12, 20 μg/ml concentrations of 5-FU and OSA NPs- 5-FU in the A375 cell line; AO/EtBr stained A375 cells: **B** - control (untreated cells), **C** - cells treated with 5-FU (4 μg/ml), **D** - 5-FU (20 μg/ml), **E** - OSA NPs- 5-FU (4 μg/ml) and **F** - OSA NPs- 5-FU (20 μg/ml).

*In vitro*, anticancer activity was performed in A375 cells (human melanoma cells) to determine the percentage of dead cells after treatment with 5-FU and OSANP@ 5-FU in Figure 5A. The cell-killing efficiency of OSANP@ 5-FU at higher concentrations (20 μg/ml) was found to be 76.23 %, whereas that of 5-FU was 27.6 %, respectively. The 4 and 20 μg/ml concentrations of 5-FU and OSANP@ 5-FU were assessed by a live/dead assay. The dead cells appeared red by uptaking EtBr due to poor membrane integrity, and live cells appeared green. Almost all are alive in control, and images were taken at 10× magnification in a fluorescent microscope (Figure 5B). Figures 5C and 5D show the 4 and 20 μg/ml of 5-FU treated cells showing 15±1 and 28±2 % dead cells. Figures 5E and 5F show the OSA NPs- 5-FU treated with 4 and 20 μg/ml concentration causing 31±2 and 78±2 % cell death. In the A375 cells, apoptotic cell death was characterized by the observation of typical apoptotic bodies and membrane blebbing. The OSA NPs encapsulated 5-FU showed a 2.5 times higher death rate compared to 5-FU.

### Scratch assay

**Figure 6.**
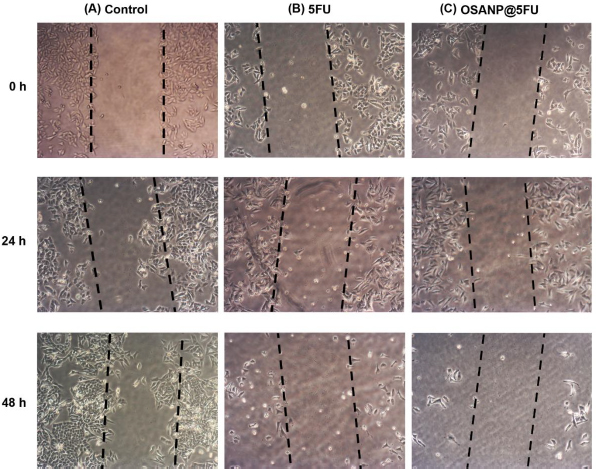
A375 cell migration rate transversely the scratches after treatment for 0 h 24 and 48 h. **A** - control (untreated cells), cells treated with 20 μg/ml doses of **B** - 5-FU and **C** - OSANP@ 5-FU.

The anticancer activity of the 5-FU and OSANP@ 5-FU was evaluated using a scratch assay to observe cell migration within the scratches on each plate. A375 cells were treated with a high concentration (20 μg/ml) of 5-FU and OSANP@ 5-FU for 24 and 48 h (Figure 6). The cells' migration capability was assessed by measuring the area (area = length × width) using an inverted phase contrast microscope. The area of the control at 0 h is 45519.9 cm^2^, and it was reduced to 28997.08 cm^2^ after 48 h. This shows significant migration of cells in control. 5-FU-treated cells prevented the majority of cells from migrating through the scratch, whereas the area was 50251.9 cm^2^ at 0 h, but it subsequently increased to 82097.6 cm^2^. The cells treated with OSANP@ 5-FU exhibited a low migration rate compared to those treated with 5-FU alone. The area of OSANP@ 5-FU at 0 h is 41936.3 cm^2^, and it becomes 110115.6 cm^2^ after 48 h. As expected, the sensitive cells started dying. These findings again support the inference that OSANP@ 5-FU possesses superior potential anticancer properties than free 5-FU.

## Conclusion

In this study, OSA polymer was synthesized and nanoformulated to improve the efficacy of 5-FU in cancer treatment. The predominant S2→S0 transitional data of diketo form of 5-FU obtained from steady-state emission spectra and dynamic light scattering observation confirmed the successful encapsulation of 5-FU in OSA NPs with an average hydrodynamic diameter of 226.6 nm with a polydispersity index value of 0.363. The encapsulation efficiency was 68 % and provided a sustained discharge or release profile by the Fickian diffusion method (Korsmeyer-Peppas model). OSANPs provide a better release in an acidic medium (pH-2.2), suggesting OSANPs can be used as a pH-receptive smart drug distribution vehicle. The safety of OSANPs as a drug delivery system was proven by cell viability evaluation using V79 cell lines. The study utilized scratch and live/dead assays to assess the anticancer efficacy, finding that OSANPs encapsulating 5-FU demonstrated heightened anticancer activity against the A375 cell line. Hence, the improved photophysical stability of 5-FU, along with enhanced biocompatibility, anticancer efficacy, and pH-sensitive sustained release, positions OSANP@ 5-FU as a promising advancement in cancer treatment.
